# Genetic Connectivity of the Moth Pollinated Tree *Glionnetia sericea* in a Highly Fragmented Habitat

**DOI:** 10.1371/journal.pone.0111111

**Published:** 2014-10-27

**Authors:** Aline Finger, Christopher N. Kaiser-Bunbury, Chris J. Kettle, Terence Valentin, Jaboury Ghazoul

**Affiliations:** 1 Environmental Systems Science, ETH Zürich, Zürich, Switzerland; 2 Department of Biology, Technical University of Darmstadt, Darmstadt, Germany; 3 National Park Authority, Victoria, Mahé, Seychelles; Central China Normal University, China

## Abstract

Long-distance gene flow is thought to be one prerequisite for the persistence of plant species in fragmented environments. Human influences have led to severe fragmentation of native habitats in the Seychelles islands, with many species surviving only in small and isolated populations. The endangered Seychelles endemic tree *Glionnetia sericea* is restricted to altitudes between 450 m and 900 m where the native forest vegetation has been largely lost and replaced with exotic invasives over the last 200 years. This study explores the genetic and ecological consequences of population fragmentation in this species by analysing patterns of genetic diversity in a sample of adults, juveniles and seeds, and by using controlled pollination experiments. Our results show no decrease in genetic diversity and no increase in genetic structuring from adult to juvenile cohorts. Despite significant inbreeding in some populations, there is no evidence of higher inbreeding in juvenile cohorts relative to adults. A Bayesian structure analysis and a tentative paternity analysis indicate extensive historical and contemporary gene flow among remnant populations. Pollination experiments and a paternity analysis show that *Glionnetia sericea* is self-compatible. Nevertheless, outcrossing is present with 7% of mating events resulting from pollen transfer between populations. Artificial pollination provided no evidence for pollen limitation in isolated populations. The highly mobile and specialized hawkmoth pollinators (*Agrius convolvuli* and *Cenophodes tamsi*; Sphingidae) appear to promote extensive gene flow, thus mitigating the potential negative ecological and genetic effects of habitat fragmentation in this species. We conclude that contemporary gene flow is sufficient to maintain genetic connectivity in this rare and restricted Seychelles endemic, in contrast to other island endemic tree species with limited contemporary gene flow.

## Introduction

In fragmented landscapes the degree of genetic exchange may be important for species to maintain their genetic diversity, and subsequently ensure their long-term survival. Negative genetic effects, such as inbreeding depression and loss of genetic diversity, have been observed to follow population isolation and restricted gene flow [1]. Species with capacities for long-distance gene flow are likely to be less prone to population isolation following habitat fragmentation compared to species with limited gene flow [2], [3]. Wind pollinated tree species are thought to be less vulnerable to the effects of habitat fragmentation as pollen can be transported over long-distances [4], [5]. Similarly, mobile pollinators have been shown to transport pollen over extensive distances (up to tens of kilometres), thus ensuring genetic connectivity between populations that are geographically disjunct [2], [6]–[9]. Even single isolated trees can act as pollen donors or sinks, demonstrating the potential for insect pollinated tree species to maintain gene exchange across fragmented landscapes [6], [10]–[12].

Habitat fragmentation is often also accompanied by the spread of invasive plant species which have the potential to compete with native plants for pollinators [13], [14]. While habitat fragmentation negatively affects pollination success and reproductive output of both generalist and specialist pollinated plant species to a similar degree [3], [15], [16], tree species with specialised pollinators might be less vulnerable to competition for pollinators from invasive plants on account of the tight coupling of the mutualistic relationship between plant and pollinator [16], [17]. Such factors could be especially important in island systems prone to introduction of invasive plant species [18], [19]. We propose to test this hypothesis on *Glionnetia sericea*, an endangered endemic tree of Mahé, the main island of the Seychelles archipelago. Populations of *G. sericea* have been reduced to small scattered patches surrounded by forests that are heavily dominated by alien invasive plants. Other studies on the Seychelles and elsewhere have demonstrated that under such circumstances native isolated plant populations can become vulnerable to loss of genetic diversity and inbreeding [20]–[22]. Thus it is not clear whether *G. sericea* is able to maintain extensive pollen flow due to its hawkmoth pollinators and whether apparently isolated *G. sericea* populations will maintain among-population pollen flow, and as a result will maintain high genetic diversity.

The aim of this study is to understand the variation in mating system in a species which is expected to have long-distance pollen-mediated gene flow and a naturally patchy distribution. We evaluate historical and contemporary gene flow among remnant populations of *G. sericea* to explore whether the species has the potential to survive in relict populations, following habitat fragmentation. We assume that the current adult individuals pre-date recent fragmentation. We therefore address the following questions using adult (historical gene flow/pre-fragmentation), and juvenile and seed (contemporary gene flow/post-fragmentation) cohorts: (i) What is the extent of gene flow between discrete *G. sericea* adult populations? (ii) Does contemporary pollen or seed dispersal prevent genetic structuring between patches? (iii) Is *G. sericea* pollen limited in fragmented sites? We discuss the relevance of the results from the perspective of conservation of endangered island plant species.

## Materials and Methods

### Study species and populations

The rare tree *Glionnetia sericea* (Rubiaceae) is endemic to the Seychelles archipelago. It is found on two islands, Mahé and Silhouette, and the total population size is estimated at fewer than 2500 individuals (Mahé and Silhouette [23]). *Glionnetia sericea* is representative of a plant community occurring in virgin forest remnants but is mostly found on inselbergs (granitic outcrops) at altitudes of about 450 m–900 m. These habitats were patchily distributed even before human colonisation. Nevertheless, current populations of *G. sericea* have been reduced in size by extensive deforestation during the 20^th^ century [24], and many populations might have been lost altogether. Subsequent invasion of this habitat zone by alien invasive species such as *Cinnamomum verum*, *Psidium cattleianum*, *Syzygium jambos, Falcataria moluccana*, and *Alstonia macrophylla* have further isolated remaining populations.

Known relict populations of *G. sericea* vary from extremely small (1–14 individuals) to relatively large and more continuous (several 100 individuals). The persistence of these small and fragmented communities is threatened by habitat degradation, increasing duration and frequency of drought due to global warming, and competition with invasive species [25]. Inselbergs though provide important refugia for *G. sericea* due to harsh environmental conditions (very thin acidic soils and xerophytic conditions), which invasives appear less easily to tolerate [26].

*Glionnetia sericea* is a small to medium sized, slow growing tree (up to about 8 m), flowers are visited by two potential pollinator species, the hawkmoths *Agrius convolvuli* and *Cenophodes tamsi* which have long probosces adapted to access the nectar produced at the bottom of long narrowly tubular corollas [19]. The protandrous hermaphrodite flowers are approximately 10cm long and white in colour during the male stage, turning red for the female stage. The flowers are presented terminally, slightly protruding from the dense foliage of the canopy, and are arranged in inflorescences with up to 10 receptive flowers. Anthesis of the nocturnal flowers occurs with sun set, and each sexual stage lasts for approximately 24 hours. Flowering typically, but not exclusively, occurs during the rainy season (October–April) and fruiting during the dry season (May–September), mostly synchronously among sites. Seeds are small (about 2 mm long and 1 mm wide), light and unwinged. We expect seeds to be mainly dispersed by gravity but due to their small size and weight long-distance dispersal by wind may be possible Dispersal by animal was never recorded and is unlikely because *G. sericea* has non-fleshy fruits.

**Figure 1:**
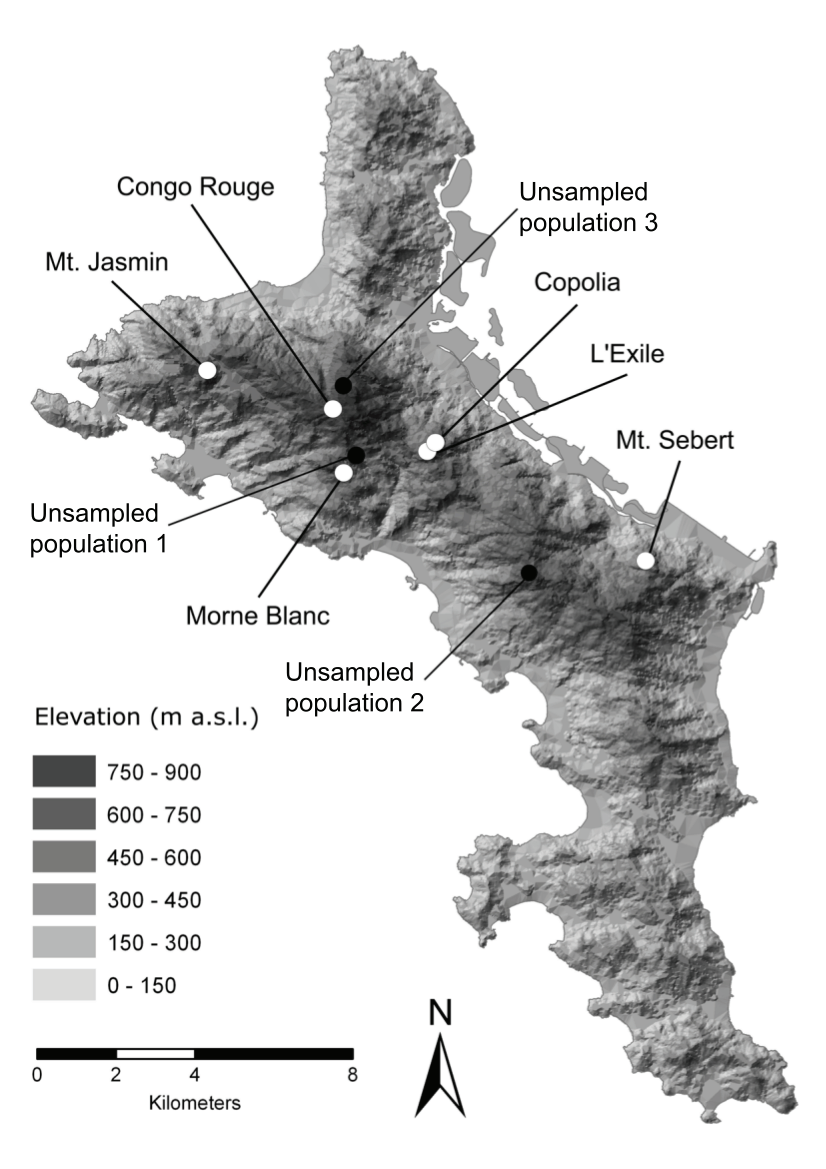
Map of the Seychelles main Island Mahé, with sampled and known unsampled *Glionnetia sericea* populations.

We sampled leaf material for genetic analysis from adults, juveniles (seedlings and saplings with an estimated age range of 1 to 10 years) of six sites and seeds of five sites. Permission for these collections was received from the Seychelles Bureau of Standards on Mahé. The total number of sampled adults (181) represents 13% of the total estimated population size on Mahé. Sites included six from nine known sites on Mahé, including four inselberg habitats: Mt. Sebert: <20 trees (of which 11 sampled); Copolia: about 50 trees (of which 28 sampled); L’Exile: about 100 trees (of which 34 sampled); Morne Blanc: seven trees all of which were sampled and two mist forest sites at Mt. Jasmin and Congo Rouge (each about 150 trees, of which 58 and 45 sampled, respectively) (Fig. 1 and Table 1). It is possible that we might have missed some small trees in the small populations, though our survey of trees was thorough. The known unsampled sites (one close to Copolia, one east of Mt. Sebert on the opposite side of the mountain ridge, and one at 850 m elevation close to the highest peak of the island) were inaccessible at the time of collections. The sampling scheme sought to encompass the whole range of each sampled population. The population range varied slightly between populations, and the furthest distance between two sampled trees within populations was 208 m for Mt. Sebert, 103 m for Mt. Jasmin, 171 m for Copolia, 60 m for L’Exile, 69 m for Congo Rouge and 28 m for Morne Blanc. Distance between populations ranged between 12 km between Mt. Sebert and Mt. Jasmin to less than 1 km between Copolia and L’Exile.

**Table 1:** Genetic variability of 10 microsatellite loci estimated for all populations of *Glionnetia sericea*.

POPID	n			*H*_O_			*H*_E_			*R*_S(5)_			P_A_	*F*_IS_		
	Ad	Jv	Sd	Ad	Jv	Sd	Ad	Jv	Sd	Ad	Jv	Sd	Ad	Ad	Jv	Sd
MS	11	12	172	0.50(±0.09)	0.38(±0.07)	0.47(±0.08)	0.49(±0.09)	0.47(±0.07)	0.49(±0.09)	3.07(±0.47)	3.36(±0.55)	2.89(±0.38)	2	ns	0.24**	0.03*
MJ	58	41	79	0.45(±0.08)	0.47(±0.08)	0.47(±0.07)	0.52(±0.09)	0.51(±0.09)	0.51(±0.08)	3.19(±0.49)	3.21(±0.51)	3.01(±0.41)	4	0.13***	0.08*	0.11**
CO	28	30	233	0.44(±0.08)	0.47(±0.07)	0.41(±0.07)	0.48(±0.09)	0.40(±0.09)	0.49(±0.09)	3.04(±0.43)	2.98(±0.34)	3.06(±0.43)	3	0.09*	ns	0.18***
LE	34	43	21	0.56(±0.06)	0.51(±0.04)	0.48(±0.03)	0.57(±0.05)	0.57(±0.05)	0.55(±0.05)	3.09(±0.35)	3.12(±0.31)	2.92(±0.28)	1	ns	ns	0.15**
CR	45	23	90	0.39(±0.08)	0.36(±0.07)	0.38(±0.08)	0.46(±0.10)	0.46(±0.09)	0.47(±0.10)	2.99(±0.48)	2.90(±0.42)	2.95(±0.50)	6	ns	0.16**	0.20***
MB	7	29	0	0.43(±0.12)	0.38(±0.08)	NA	0.40(±0.10)	0.40(±0.09)	NA	2.63(±0.50)	2.47(±0.37)	NA	2	ns	ns	NA
**Total**	**181**	**178**	**595**	**0.46** **(±0.03)**	**0.43** **(±0.03)**	**0.44** **(±0.03)**	**0.48 ±(0.04)**	**0.49** **(±0.03)**	**0.50** **(±0.04)**	**3.00** **(±0.18)**	**3.01** **(±0.16)**	**2.96** **(±0.17)**		0.07***	0.09***	0.13***

Abbreviations: Ad: Adults; Jv: Juveniles; Sd: Seeds; n: number of genotyped individuals; *H*_O_: observed heterozygosity; *H*_E_: expected heterozygosity; *R*_S_: Allelic richness, based on (five) diploid individuals; *P*_A_: total number of private alleles (mean frequency); *F*_IS_: Inbreeding coefficient; ± SE. * = p<0.05, ** = p<0.01, *** = p<0.001, ns = not significant. MS = Mt. Sebert, MJ = Mt. Jasmin, CO = Copolia, LE = L’Exile, CR = Congo Rouge, MB = Morne Blanc.

### Sampling and genetic analysis

In 2009 we collected leaf material for DNA extraction from *G. sericea* adults, seedlings and seeds. To increase the power of our analyses we only included in our final data set individuals with a minimum of 8 typed loci. We thus analysed 181 adults and 178 juveniles (between 20 cm and 1 m height) at six sites and 595 seeds (from 24 mother trees) at five sites, see Table 1. Leaf material was immediately dried and stored in silica gel. DNA was extracted from the leaves using the QIAGEN DNeasy 96 Plant Kit, following the manufacturer’s protocol. All samples were screened at a total of ten nuclear microsatellite loci, details of which are described in [27]. Fragment analysis was conducted using an ABI3730 sequencer and genotyped using Genemapper 3.5 software (Applied Biosystems). There was no evidence for linkage disequilibrium for any pair of loci and no evidence of null alleles after Bonferroni corrections, see [27].

#### Assessment of genetic diversity and inbreeding

Number of alleles (*N*_A_), observed and expected heterozygosities (*H*_O_, *H*_E_), and the number of private alleles (*P*_A_) were calculated using GenAlEx 6.5 [28]. Allelic richness (*R*_S_) was calculated using FSTAT 2.9.3.2 [29]. Overall species inbreeding coefficients (*F*_IS_) and differentiation (*F*_ST_), population specific *F*_IS_ values (using 1023 permutations), and a hierarchical genetic variance analysis (AMOVA) to examine the organisation of overall genetic diversity, were calculated with Arlequin 3.5 [30]. For *F*_IS_ calculations we defined populations according to their contemporary geographic locations (6 populations) even though we cannot be sure that populations may be connected though gene flow. Nevertheless, calculations based on 6 populations should avoid a potential Wahlhund effect caused by ignoring sub-structuring in populations.

#### Assessment of genetic structure over the species range

Since we did not have an exhaustive sample size and no *a priori* rationale for defining populations we tested for the presence of geographical groupings of related samples by applying a Bayesian cluster analysis to all individuals using the software STRUCTURE v2.3.4 [31]. The batch run function was used to carry out a total of 100 runs, ten each for one to ten clusters (K1 to K10). For each run the burn-in and simulation length was 20,000 and 50,000, respectively. Since the log probability values for the different K values have been shown to be of little reliability in other cases, the more refined ad hoc statistic ΔK based on the rate of change in the log probability of data between successive K values [32] was used. It is calculated as ΔK = ([mL(K+1)–2 mL(K)+mL(K–1)])/sdL(K), where L(K) is the logarithm of the probability that K is the correct number of clusters, m is the mean and sd is the standard deviation.

Genetic isolation by distance was tested for by use of a Mantel permutation procedure which was executed in GenAlEx 6.5. For this analysis we used the linearized pairwise *F*_ST_ values and the log distance between populations.

#### Estimating realised gene flow using paternity analysis

Using multilocus genotypes (ten loci) of 595 seeds we applied a maximum likelihood exclusion analysis in CERVUS 3.0, to assign the most likely candidate fathers, given known mothers [33], [34]. Simulations of paternity were run using the allele frequencies of all adult reproductive trees and the following settings: 10000 cycles; minimum number of loci typed 8, known mothers; all sampled adults across the species range were set as candidate fathers for seeds; 1% for proportion of loci mistyped, and 92% for proportion of loci genotyped. We collected a high proportion of individuals within populations (about 80%, for some populations up to 100% of all known trees) and we assume that most pollination events should occur within populations with fewer long-distance mating events. Nevertheless, as we did not sample all potential parents and may have overlooked trees within populations due to the difficult terrain, we set the proportion of candidate parents sampled at 50%. Assignment was based upon the 95% (strict) and 80% (relaxed) confidence level of the critical LOD score.

Selfing rates were calculated as the number of cases where paternity analysis determined the father as the same tree as the known mother. Given that the seeds are collected directly from known mothers it is highly unlikely that the CERVUS analysis fails to detect all selfed individuals and unlikely that the unassigned seeds are selfed. Therefore we estimated the selfing rate as the proportion of selfed seeds to the total number of analysed seeds (595). Additionally, mating system was calculated using the software MLTR 3.2 [35], to verify results obtained from direct observations in the CERVUS paternity analysis. Seed gene frequencies and known mother trees were used for the analysis and mating system estimates and standard errors are based on 100 bootstraps.

The level of outcrossing and correlated paternity was estimated by a correlated-matings model implemented in MLTR 3.2 using the genotypes of the 595 seeds and their respective mothers. The software estimates the correlation of outcrossed paternity within progeny arrays (multilocus estimator).

### Experimental pollinator exclusion

To test for the potential effects of pollinator limitation, and the relative benefits of pollen dispersal within and between populations, we conducted the following pollination experiments in 2009. For 16 trees at Copolia and 7 trees on Mt. Sebert, inflorescences, each with about 30 flowers at bud stage, were enclosed within Delnet pollination bags. In total 25 bags were used on Mt. Sebert, and 35 bags on Copolia. Within each bag the following treatments were applied, at Mt. Sebert: Selfing, using pollen sourced from flowers of the same tree; Within-population crosses using mixed pollen sourced from four or five other trees at Mt. Sebert; Between-population crosses using a random mix of pollen donors (four or five trees) from the Mt. Jasmin population; Between-population crosses using a random mix of pollen donors (four or five trees) from the Congo Rouge population; and unmanipulated (non-pollinated) flowers as complete pollen exclusion. On Copolia the same treatments were applied except for Between-population crosses which used pollen from the Congo Rouge population. As a control we determined seed set of unmanipulated flowers outside pollination bags, which were therefore openly accessible to pollinators. Pollen was transferred using a fine paint brush to viable stigmas of flowers. The period of stigma viability had been previously determined on other flowers using the Peroxtesmo Ko test [36]. Flowers were individually labelled to distinguish between treatments. The pollination bags were retained on trees until the fruits were ripe for collection. Mature seeds proved to be either viable (developed and full) or non-viable (developed but empty). To determine fitness values for each pollination treatment we recorded fruit set (developed fruits as a proportion of treated flowers) and seed set (viable seeds as a proportion of all developed seeds).

#### Pollination analysis

Statistical analyses of the pollinator exclusion experiment were carried out in R, version 2.10.1 [37]. We used generalized linear mixed-effect models (GLMM) with a binomial error distribution to analyse fruit and seed set. GLMM can account for the nested experimental design, and we included bags nested in trees as a random effect in the model [38]. We applied the lmer function from the lme4 library [39]. Due to the different number of flowers and fruits per tree and treatment the data for seed set were unbalanced. Our analysis is robust for unbalanced data by using the “cbind” function which calculates fruit and seed set weighted by sample sizes [38]. We ran two sets of models: a main effect model with treatment as fixed effect to determine the level of selfing, and a full model with treatment (only within- and between-population crosses) and tree ID as fixed effects to look at the effects of between population crosses. Model selection was based on the Akaike Information Criterion (AIC) and best-fitted models were determined by ΔAIC <2. We ran two models a full-factorial model including all treatments and a smaller, nested model in which we removed the treatments selfed and non-pollination to compare effects of within- and between-population crosses.

If possible, ripe and full fruits were collected for the controls. Often the ripe fruits were already opened and had released some or even most of their seeds, so that the number of seeds collected per tree was summed up independently from the number of collected fruits and taken as one sample for each tree. Thus, only 19 samples could be collected as a control group for Copolia and 9 for Mt. Sebert. We only sampled developed fruits for the control group and so we compared seed set values of the control group with only developed fruits of the other treatments. To test for significant differences the 95% confidence interval was calculated for the mean seed set values.

## Results

### Genetic diversity and differentiation

#### Genetic diversity and inbreeding of adult, juvenile and seed cohorts for 10 loci

At the species level the ten loci yielded between three and 15 alleles, with a total number of 82 alleles. To make sure that null alleles have no effect on our analyses we additional calculated *F*_IS_ using INEST 2.0 [40] which accounts for null alleles. As our results were qualitatively consistent with FSTAT results presented here (data not shown) we only present the FSTAT analysis. A comparison of genetic diversity over all loci and populations is given in Table 1. A comparison of the three groups (adults, juveniles and seeds) in FSTAT resulted in no significant difference of *H*_E_, *H*_O_, *F*_IS_ or allelic richness between groups, using a two-sided test of significance after 1000 permutations. Thus, there is no significant difference between historical and contemporary genetic diversity in *G. sericea*.

#### Genetic differentiation of adult and juvenile cohorts

**Figure 2:**
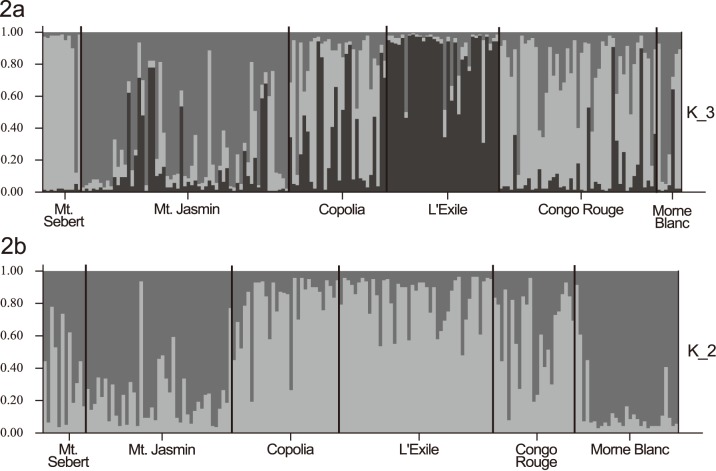
Bayesian structure analysis of *Glionnetia sericea* with the STRUCTURE software. Bars represent individual *G. sericea* adult individuals with their assignment proportions (y axis) to the different clusters/genetic groups. Performing the analysis for K_3 (three different grey shades), representing three genetic clusters (**a**). STRUCTURE analysis for *G. serivea* juveniles, performing the analysis for K_2 (two different grey shades), representing two genetic clusters (**b**).

The AMOVA analysis revealed an overall mean pairwise genetic distance *F*_ST_ value of 0.09, 0.07, 0.12 (all p<0.001) for adults, juveniles and seeds, respectively. *F*_ST_ was significantly different among all populations (Table 2). The geographically most distant populations Mt. Jasmin and Mt. Sebert (0.14, p<0.05; 13 km) obtained similar values as geographically closer sites, e.g. Congo Rouge and L’Exile (0.13, p<0.05; about 3 km). Morne Blanc and L’Exile showed generally high pairwise *F*_ST_ values despite being geographically close to neighbouring populations. The STRUCTURE analysis identified three distinct genetic clusters (showing the highest ΔK value at K3- results not shown) as the most likely solutions indicating a delineation of L’Exile and Mt. Sebert populations from all other individuals (see Fig. 2a). For juvenile cohorts the highest likely number of clusters has been calculated to be two (showing the highest ΔK value at K2- results not shown), seemingly clustering Copolia, L’Exile and Congo Rouge populations and Mt. Sebert, Mt. Jasmin and Morne Blanc populations (see Fig. 2b). These results therefore show no stronger genetic differentiation for contemporary post-fragmentation (juveniles) compared to historic pre-fragmentation samples (adults).

**Table 2:** Pairwise *F*_ST_ values between adult (Ad; above the diagonal) and juvenile populations (Jv; below the diagonal), * = p<0.05, with adjusted nominal level (0.003) for multiple comparisons.

Jv\Ad	MS	MJ	CO	LE	CR	MB
MS	0	0.14*	0.10*	0.17*	0.09*	0.12*
MJ	ns	0	0.05*	0.09*	0.07*	0.06*
CO	ns	0.06*	0	0.07*	0.04*	0.08*
LE	ns	0.06*	0.03*	0	0.13*	0.15*
CR	ns	0.05*	0.03*	0.07*	0	0.05*
MB	ns	0.09*	0.14*	0.13*	0.14*	0

MS = Mt. Sebert, MJ = Mt. Jasmin, CO = Copolia, LE = L’Exile, CR = Congo Rouge, MB = Morne Blanc.

As the genetic clusters are not in accordance to the geographic location of the actual populations we did not merge *G. sericea* adults into these three groups for further analyses, as doing so would bring together individuals from distant populations, which does not seem to be biologically sensible. We rather think that the STRUCTURE analysis demonstrates high gene flow rates between populations and therefore decided to keep individuals in six populations, according to their geographic location.

The Mantel test showed no significant result (R^2^ = 0.0087, p = 0.39) suggesting no genetic isolation by distance for *G. sericea* populations.

#### Contemporary gene flow and mating system

A paternity analysis conducted for the seeds, given known mothers, could assign 28% (169 out of 595 seeds) with 95% confidence, 55% (326 seeds) with 80% confidence. Results for the assignments with 95% and 80% confidence are presented in Table 3a and b, respectively. Overall, out of the 326 assigned seeds 56% mating events resulted from matings within populations (excluding selfed seeds) and 17% from gene flow between populations. Selfing rates over all populations were 15% (91 out of 326 seeds), ranging from 5% in L’Exile to 38% in Congo Rouge. Results for the 95% confidence showed similar within population mating events (55%) and fewer between population matings (7%). Mating system analysis of seeds, using the MLTR software, revealed similar selfing rates to the CERVUS paternity analysis with rates ranging between 5% in L’Exile and 39% in Congo Rouge, for comparison see Table 3a and b. The outbreeding analysis in MLTR suggests relatively high outbreeding rates (up to 0.98 for Mt Sebert) and also a high correlated paternity for some populations (e.g. 51% of all Congo Rouge seeds are likely to share the same father), see Table 4.

**Table 3:** Gene flow and selfing rates for *Glionnetia sericea* populations obtained from a paternity analysis implemented in CERVUS, assignment rates based on 95% (a) confidence and 80% confidence (b).

a.											
Population	N total	N assignments	Fathers within pops			Fathers outside pops			Selfed		Sefing rate MLTR
	Seeds		#	Prop	Poll Dist in m	#	Prop	Poll Dist in m	#	Prop to ntotal	Prop (SD)
MS	172	59	41	0.69	2.00 (37.63)	7	0.12	8636.97(403.82)	11	0.06	0.11 (0.10)
MJ	79	14	10	0.71	24.72 (16.54)	0	0.00		4	0.05	0.13 (0.12)
CO	233	55	32	0.58	7.25 (9.08)	2	0.04	5819.91(45.15)	21	0.09	0.18 (0.13)
LE	21	6	5	0.83	40.29 (3.39)	0	0.00		1	0.05	0.05 (0.27)
CR	90	35	5	0.14	9.96 (0.00)	2	0.06	3539.47 (14.47)	28	0.31	0.39 (0.18)
**All**	**595**	**169**	**93**	**0.55**	**8.57 (35.60)**	**11**	**0.07**	**7830.19** **(2817.05)**	**65**	**0.11**	
**b.**											
MS	172	100	64	0.64	1.88 (36.88)	18	0.18	8627.32(5580.54)	18	0.10	0.11 (0.10)
MJ	79	35	22	0.63	20.96 (11.74)	7	0.20	3539.21(230.02)	6	0.08	0.13 (0.12)
CO	233	126	77	0.61	7.25 (45.94)	17	0.14	2777.34(3280.61)	32	0.14	0.18 (0.13)
LE	21	10	6	0.60	38.60 (5.24)	4	0.40	2665.79 (3280.61)	1	0.05	0.05 (0.27)
CR	90	55	13	0.24	9.96 (5.24)	8	0.15	3516.52(799.04)	34	0.38	0.39 (0.18)
**All**	**595**	**326**	**181**	**0.56**	**7.25 (35.02)**	**54**	**0.17**	**5796.47** **(5580.54)**	**91**	**0.15**	

As a comparison to the CERVUS analysis selfing rates for seeds were also obtained with the MLTR software. MS = Mt. Sebert, MJ = Mt. Jasmin, CO = Copolia, LE = L’Exile, CR = Congo Rouge. # = Numbers, Prop = Proportions to the number of assignments, Poll Dist = The median of the observed pollination distance with the Interquartile Range in brackets.

**Table 4:** Population-level estimates of mating system and correlated paternity implemented in MLTR 3.2, standard deviations are presented in brackets.

Population	Multilocus outcrossing rate (tm)	Correlated paternity (rp)
MS	0.98 (0.10)	0.21 (0.24)
MJ	0.87 (0.12)	0.27 (0.18)
CO	0.82 (0.18)	0.32 (0.08)
LE	0.95 (0.27)	0.37 (0.03)
CR	0.61 (0.18)	0.51 (0.14)

MS = Mt. Sebert, MJ = Mt. Jasmin, CO = Copolia, LE = L’Exile, CR = Congo Rouge.

**Figure 3:**
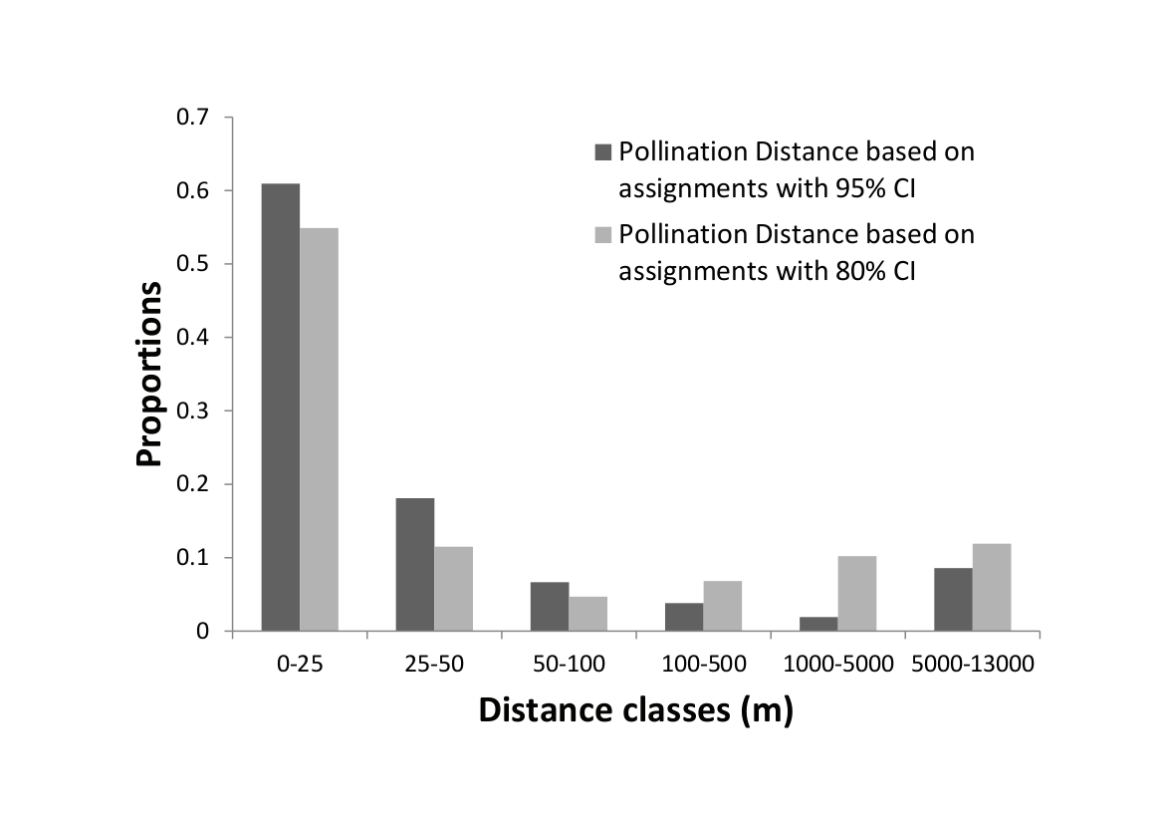
Frequency distributions of realised pollen flow distances in *Glionnetia sericea* populations, calculated from n = 105 juveniles and parent pairs for the 95% CI (dark grey) and n = 235 for the 80% CI (light grey).

**Figure 4:**
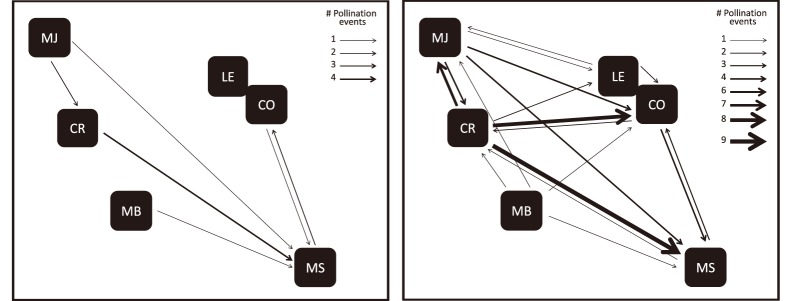
Long-distance (between-population) realised pollen flow directions for *Glionnetia sericea* based on (a) assignments with 95% CI and (b) assignments with 80% CI. The arrows give the pollen flow directions and the thickness represents the observed numbers of pollination events.

Realised pollen dispersal ranged between 0.34 m to 12.14 km, with nearly half of all seeds resulting from mating events of less than 20 metres (median = 18.22 m, Interquartile Range = 352.37). Within population pollen dispersal ranged from 0.34 m to 481.39 m and was mostly less than 10 m (median = 7.25, Interquartile Range = 35.02), between population pollen dispersal ranged from 160.71 m to 12.14 km and was mostly less than 6 km (median = 5796.47 m, Interquartile Range = 5580.54), see Table 3a and b. A pollen frequency distribution (using geographic distances between parent pairs) based on assignments of 95% confidence and 80% confidence is shown in Fig. 3. Based on the assignments at 95% confidence the paternity analysis shows that Mt Sebert mainly acts as a pollen sink, whereas Congo Rouge is a pollen source to many populations. Based on the assignments at 80% multidirectional pollen flow is present among populations (see Fig. 4a and b).

### Pollen exclusion and between population pollination crossing experiment

#### Fruit and seed set at Copolia and L’Exile

Fruit set was not significantly different when comparing between-population (0.31±0.06) and within-population crosses (0.52±0.05; z = −1.857, p = 0.06). Highest fruit set was obtained for the open controls (0.84±0.02) and this result was significantly higher than between-population (z = 5.275, p<0.001) and within-population crosses (z = 3.942, p<0.001), see Table 5a and b.

**Table 5:** 

a.							
Pops	All fruits	Treatments					
		SE	WI	BW	BW2	NO	OP
MS	Mean Seed set	0.23±0.04	0.38±0.04	0.36±0.04	0.35±0.06	0.05±0.01	-
	# Fruits	78	80	81	37	517	-
	# Used fruits	75	71	78	33	495	-
	Fruit set	0.44±0.07	0.60±0.07	0.63±0.09	0.65±0.11	0.18±0.03	0.76±0.03
	# Fruits	82	86	87	41	547	87
	# Trees	6	7	7	5	7	3
	# Bags	21	22	18	13	25	-
CO	Mean Seed set	0.28±0.04	0.26±0.04	0.20±0.04	ND	0.01±0.00	-
	# Fruits	89	81	96	ND	797	-
	# Used fruits	84	75	89	ND	745	-
	Fruit set	0.46±0.07	0.52±0.05	0.31±0.06	ND	0.09±0.02	0.84±0.02
	# Fruits	95	80	102	ND	866	231
	# Trees	14	14	14	ND	16	9
	# Bags	28	23	27	ND	35	-
	Full fruits	SE	WI	BW	BW2	NO	OP
MS	Mean Seed set	0.58±0.04	0.62±0.03	0.66±0.02	0.58±0.04	0.66±0.03	0.56±0.05
	Used fruits	30	43	42	20	36	9
	–95% CI	0.50	0.56	0.61	0.51	0.61	0.45
	+95% CI	0.66	0.67	0.70	0.66	0.72	0.66
CO	Mean Seed set	0.63±0.03	0.63±0.04	0.70±0.04	ND	0.65±0.06	0.63±0.04
	Used fruits	36	31	26	ND	15	19
	–95% CI	0.58	0.56	0.62	ND	0.53	0.57
	+95% CI	0.68	0.70	0.77	ND	0.76	0.69

b.							
**MS\CO**		**Fruit set**					
SE		-	ns	ns	ND	***	***
WI		*	-	ns	ND	***	***
BW		Ns	ns	-	ND	***	***
BW2		**	ns	ns	-	ND	ND
NO		***	***	***	***	-	***
OP		**	ns	ns	ns	***	-
**MS\CO**		**Seed set**					

**a.** Summary table of results of pollination experiments. Fruit set and seed set are given in mean proportions ± SE. SE = Selfed individuals, WI = Within population crosses, BW = Between population crosses with individuals from Mt. Jasmin, BW2 = Between population crosses with individuals from Congo Rouge, NO = Bagged individuals with no hand pollination, OP = Controls. Fruit set is defined as the proportion of flowers that developed into fruits. Seed set is defined as the proportion of developed seeds per fruit. MS = Mt. Sebert, CO = Copolia. ND = not done. All fruits: all collected fruits for seed and fruit set experiments; full fruits: A subset of collected fruits that were still closed and thus haven’t lost any seeds.

**b.** Significant differences in fruit and seed set for the different treatments. * = p<0.05; ** = p<0.01; *** = p<0.001. Values for Copolia are above and those for Mt. Sebert below the diagonal.

The difference in seed set (as a proportion of developed seeds) when comparing, between-population (0.20±0.04) and within-population treatments (0.26±0.04 SE; z = 0.54; p = 0.59) was not significantly different. There was no significant tree interaction effect with treatment (data not presented). The mean viable seed of the open control group was not significantly different than that from the other treatments (controls: 0.63, 95% CI 0.57–0.69; between-population: 0.70, 95% CI 0.62–0.77; within-population: 0.63, 95% CI 0.56–0.70). Viable seed set for selfed was high (0.28±0.04) and low for pollen exclusion (0.01±0.00), see Table 5a and b.

#### Fruit and seed set at Mt**.** Sebert

Fruit set from between-population (Mt. Jasmin: 0.63±0.09 SE and Congo Rouge: 0.65±0.11 SE) and within-population crosses (0.60±0.07 SE; z = 0.50, p = 0.62 and z = −0.89, p = 0.38, respectively) was not significantly different. Highest fruit set was obtained for the open controls (0.76±0.03 SE), but this result was not significantly different to between-population (Mt. Jasmin: z = 1.406, p = 0.16; Congo Rouge: z = −0.322, p = 0.75) and within-population crosses (z = 0.779, p = 0.44), see Table 5a and b.

Seed set from between-population (Mt. Jasmin: 0.36±0.04 SE and Congo Rouge: 0.35±0.06 SE) and within-population treatments (0.38±0.04 SE; z = 0.50, p = 0.62 and z = −0.89, p = 0.38, respectively) was not significantly different. There was no significant tree interaction effect with treatment (data not presented). The mean viable seed set of the control group was not significantly different from the other treatments (Controls: 0.56, 95% CI 0.45–0.66; between-population (Mt. Jasmin): 0.66, 95% CI 0.61–0.70; between-population (Congo Rouge): 0.58, 95% CI 0.51–0.66; within-population: 0.62, 95% CI 0.56–0.67). Viable seed set for the selfed treatments was high (0.28±0.04) and low for the no-pollination treatments (0.01±0.00 SE), see Table 5a and b.

## Discussion

*Glionnetia sericea* from the Seychelles illustrates the importance of long-distance gene flow for preventing loss of genetic diversity within populations in a highly fragmented habitat. Our results suggest extensive pollen flow across most populations on Mahé due to a strong flying and wide ranging hawkmoth pollinators, which prevents strong genetic differentiation between populations. Small and distant populations were not any more genetically isolated than larger or central populations. Artificial pollination experiments showed that despite human induced habitat fragmentation and high numbers of invasive plants, pollen quantity is not limited and pollen quality within population is high enough to prevent reduced reproductive outcome. We discuss our results in the context of the reproductive ecology of this species and their implications for persistence of moth pollinated plants in highly fragmented landscapes.

### Genetic diversity and inbreeding in a fragmented environment

The genetic diversity found in *G. sericea* adults does not differ between small and large populations indicating historically stable populations with no apparent genetic depletion, genetic bottlenecks or genetic drift. The only population with comparatively low genetic diversity is Morne Blanc, which comprises only nine trees all of which are small in size (based on diameter at breast height measurements; data not presented) relative to the other populations. Morne Blanc might therefore be a relatively young population derived from a recent founder event. Across all populations, genetic diversity did not differ significantly between adults, juveniles and germinated seeds, indicating no genetic erosion from adult to juvenile cohorts. Contemporary gene flow therefore seems sufficient to maintain the existing genetic diversity across generations.

Substantial inbreeding is not unusual for species that persist in small populations and have purged deleterious alleles [41]. On Mahé, for example, the endemic tree *Vateriopsis seychellarum* has highly inbred populations that currently show no obvious inbreeding depression [21]. In our study inbreeding was present in some but not all populations (large and small). Only for seed cohorts all populations were inbred but as this is the stage prior to germination we don’t know whether inbred seeds will grow into juveniles and adults. The only population that has high selfing rates and inbreeding in both seeds and juveniles is Congo Rouge. It is possible that this population has experienced reduced pollination frequency over the past few years resulting in increased prevalence of matings among related individuals. Congo Rouge is a central population of approximately 150 individuals, so not obviously geographically isolated (nor historically genetically isolated as shown from our data).

For other populations, higher prevalence of inbreeding among seeds compared to juveniles or adults implies higher mortality of inbred individuals during the course of development. Selection against inbred offspring has already been observed for another endemic Seychelles tree *Medusagyne oppositifolia* where experimental pollinations resulted in greater viability of seeds and longer seedling survival of individuals derived from between-population crosses as compared to within-population crosses [20]. These results show that for very rare endemic species there is an opportunity to increase seedling performance by enhancing cross pollination either artificially or by transplanting individuals among populations to increase local genetic diversity. Such strategies need to recognise that this might undermine locally adapted traits and should therefore be undertaken with caution, though such considerations might have less weight when faced with reduced seed viability and seedling performance in existing remnant populations. Nonetheless, unlike *Medusagyne oppositifolia*, substantial gene flow implies a greater degree of resistance to genetic erosion.

### Population connectivity and the role of plant-pollinator interactions

The STRUCTURE analysis and the non-significant isolation by distance analysis suggest low genetic structuring, although significant overall *F*_ST_ and pairwise *F*_ST_ values indicate a certain degree of structure due to geographic isolation. We interpret this seemingly contradictory finding as a consequence of extensive pollen flow in combination with low seed dispersal. Moreover, most pollen is distributed among neighbouring trees (overall median pollination distance was 18.22 m), which could promote genetic differentiation between populations. Nevertheless, occasional long-distance pollen flow events appear sufficiently frequent to prevent strong genetic differentiation of populations. Low genetic differentiation between populations and even across islands in the Seychelles has also been shown in the coco-de-mer palm, *Lodoicea maldivica* [42], which must be attributed to pollination by wind as its huge seeds simply fall under the parent tree. By comparison, the formerly widespread Seychelles tree *Vateriopsis seychellarum* showed strong genetic differentiation following habitat fragmentation [21]. This tree also has poorly dispersed seed (wingless dipterocarp fruit) and is pollinated by small insects that are unlikely to have extensive pollen dispersal capacities.

Hawkmoth pollinators have been shown to move over long-distances [43], [44] even up to 10 km [45], [46]. Assuming this is also true of the hawkmoth pollinators of *G. sericea*, then pollen could potentially be exchanged among most *G. sericea* populations on Mahé, the furthest distance between populations being about 12 km. Pollination by hawkmoths of the Costa Rican tree *Pithecellobium elegans* resulted in average pollen dispersal of 142 m and a maximum distance of 350 m [47]. In this study 7% of pollination events (17% based on assignments with 80% confidence) resulted from matings between populations that had a median distance of 5.8 km and a maximum pollination distance of 12 km. Low assignment rates in our paternity analysis, however, suggest that either the sampling of adults was not as comprehensive as necessary for such fragmented populations, or low genetic differentiation and high genetic similarity between *G. sericea* individuals and populations resulted in a reduced power to assign fathers. Nevertheless, this result shows (as indicated by the STRUCTURE analysis) that long-distance pollen flow is relatively common for *G. sericea* and confirms the effectiveness of hawkmoth as long-distance pollinators. The low genetic structuring of the adult cohort across the whole island indicates extensive historic gene flow, possibly also due to small light-weight seeds that have the potential for wind dispersal. Genetic structuring does not increase in the juvenile cohort, supporting the view that contemporary gene flow is not restricted compared to past gene flow.

Compared to wind pollinated species, which can also exhibit long-distance gene flow [48]–[50], specialised insect pollinators are furthermore likely to promote targeted pollen movement as they actively seek host populations, even when these are relatively rare and isolated. Indeed, for some species even single isolated remnant insect-pollinated trees receive pollen from several distant pollen donors [2], [11], [12]. This is also the case for *G. sericea* where even one of the smallest and most remote populations Mt Sebert (comprising only 11 known adults) receives pollen from the distal populations. Thus, population fragmentation might not necessarily have negative genetic effects subject to the dispersal capabilities of the pollinators. It may be that the highest conservation concern for *G. sericea* is the maintenance of its hawkmoth pollinators. Given the considerable habitat transformation in the Seychelles over the past 100 years, including widespread loss of the native forest and its replacement with exotic invasive trees and plants in both the canopy and understorey, it is possible that the larval host plant of these moths has also been much reduced. *Agrius convolvuli* larvae are, however, known to feed on plants from a wide range of families [51] suggesting that the *G. sericea*-pollinator mutualism might be robust to such changes (though little is known of the host plants of the second pollinator *Cenophodes tamsi*). While many rare and highly fragmented plant populations in the Seychelles need urgent conservation action, our results suggest that priority should be afforded to those species that have poor pollen and seed dispersal, for which genetic rescue through artificial cross pollinations might be warranted.

### Is pollen limited in small and distant populations?

Historical and contemporary gene flow by pollen seems to have connected *G. sericea* populations over the whole of Mahé, despite habitat fragmentation. Whether this is also the case for small (as few as 14 individuals) and isolated populations is not clear. Our artificial pollination experiments could not detect any pollen limitation in either of the small or large population suggesting that the hawkmoth pollinators are sufficiently abundant so far as pollination of *G. sericea* is concerned. Our results imply that non-manipulated, open pollinated flowers develop just as many if not more fruits and seeds compared to flowers that have been hand pollinated. Further, the small and isolated population, Mt. Sebert, had similar seed and fruit set values compared to the larger population Copolia. If pollen quantity can be associated with the amount of seed and fruit set in *G. sericea* this would imply similar numbers of pollination events in both populations.

For Mt. Sebert, the geographically most isolated population, seed set and fruit set from selfed flowers were significantly lower compared to the other treatments. Conversely and contrary to expectations, selfed flowers on Copolia had similar seed and fruit set compared the within- and between-population crosses. Neither fruit set nor seed set differed significantly between the treatments within-population and between-population at the two sites, which may demonstrate that pollen quality/compatibility does not differ between populations. Nevertheless, it is worth mentioning that seed set at Copolia was much lower for between-population crosses compared to within-population crosses (with, at p = 0.06, marginal statistical significance). Copolia has been shown (e.g. in the STRUCTURE analysis) to be more isolated than the other populations and thus this lowered seed set for between-population crosses in combination with no reduced seed and fruit set for selfed flowers could hint toward potential outbreeding depression when flowers are being pollinated from distant populations. Non-pollinated flowers within bags (pollen exclusion) did produce fruits and seeds at both sites but at much lower frequencies showing that apogamy is possible but reproductive output and potentially seed viability is low. This also implies that *G. sericea* may be vulnerable to a declining pollinator visitation frequency, for example due to a behavioural or numerical change in pollinators due to habitat transformation (e.g. [52], [53]). Nevertheless, hawkmoths are opportunistic feeders [46] and some invasive plants produce plenty of nectar. Whether these invasives serve to maintain large populations of hawmoths to the benefit of *G. sericea*, or whether they compete for pollinators with *G. sericea*, as has been shown in other tropical communities of lepidopteran-pollinated species [13] has yet to be tested.

We have to account for several caveats. Fruit set, yet not seed set, was higher for the open controls in both populations indicating that the pollination bags and/or hand pollinations might have had a negative effect on fruit development. To avoid any misinterpretation of our results we used both fruit and seed set to draw conclusions of the pollination study. The between-population crosses involved the transportation of pollen from one inselberg to another with pollen stored in open tubes overnight before being applied to flowers the following day. This may have resulted in reduced pollen viability for between-population compared to within-population treatments. Finally, in field pollination experiments, unintended pollen transfer may also result in some degree of contamination within pollination bags which could explain similar responses for the within-, between-population crosses and the controls.

Despite these caveats, our study has shown that highly mobile pollinators (hawkmoths) can transport pollen effectively over relatively long-distances and that potentially wind dispersed seeds contribute to long-distance gene exchange between populations. We could also show that a possible decline in pollinator visitation frequency, which may follow habitat degradation or an increase in alien invasive plants, may decrease the species reproductive output and thus long-term population viability. So far, we could not detect strong evidence of pollen limitation even in distant populations, demonstrating a certain resistance to habitat fragmentation and/or the invasiveness of alien species of these hawkmoths species in the Seychelles. The results of this study stand in contrast to those of other recent studies on rare endemic tree species on the Seychelles that appear to be much more vulnerable to genetic differentiation and inbreeding [20], [21], with the main difference being that *G. sericea* is pollinated by hawkmoths that have the potential to disperse pollen over large distances and, crucially, among populations. This implies that conservation action should prioritise species with weakly dispersing pollination vectors.

## References

[1] Van Geert A, Van Rossum F, Triest L (2008) Genetic diversity in adult and seedling populations of *Primula vulgaris* in a fragmented agricultural landscape. Conserv Genet 9: 845–853.

[2] Dick CW (2001) Genetic rescue of remnant tropical trees by an alien pollinator. Proc R Soc Lond, Ser B: Biol Sci 268: 2391–2396. 10.1098/rspb.2001.1781 PMC1088891 11703880

[3] Ghazoul J (2005) Pollen and seed dispersal among dispersed plants. Biol Rev 80: 413–443. 16094807 10.1017/s1464793105006731

[4] Robledo-Arnuncio RJ (2011) Wind pollination over mesoscale distances: an investigation with Scots pine. New Phytol 190: 222–233. 21175640 10.1111/j.1469-8137.2010.03588.x

[5] Buschbom J, Yanbaev Y, Degen B (2011) Efficient Long-Distance Gene Flow into an Isolated Relict Oak Stand. J Hered 102: 464–472. 21525180 10.1093/jhered/esr023

[6] Lander TA, Boshier DH, Harris SA (2010) Fragmented but not isolated: Contribution of single trees, small patches and long-distance pollen flow to genetic connectivity for *Gomortega keule*, an endangered Chilean tree. Biol Conserv 143: 2583–2590.

[7] Sork VL, Smouse PE (2006) Genetic analysis of landscape connectivity in tree populations. Landscape Ecol 21: 821–836.

[8] Ahmed S, Compton SG, Butlin RK, Gilmartin PM (2009) Wind-borne insects mediate directional pollen transfer between desert fig trees 160 kilometers apart. Proc Natl Acad Sci USA 106: 20342–20347. 19910534 10.1073/pnas.0902213106 PMC2787140

[9] Nason JD, Herre EA, Hamrick JL (1998) The breeding structure of a tropical keystone plant resource. Nature 391: 685–687.

[10] Ottewell KM, Donnellan SC, Lowe AJ, Paton DC (2009) Predicting reproductive success of insect- versus bird-pollinated scattered trees in agricultural landscapes. Biol Conserv 142: 888–898.

[11] White GM, Boshier DH, Powell W (2002) Increased pollen flow counteracts fragmentation in a tropical dry forest: An example from *Swietenia humilis* Zuccarini. Proc Natl Acad Sci USA 99: 2038–2042. 11842203 10.1073/pnas.042649999 PMC122315

[12] Ismail S, Ghazoul J, Ravikanth G, Shaanker R, Kushalappa CG, et al. (2012) Does long distance pollen dispersal preclude inbreeding in tropical trees? Fragmentation genetics of *Dysoxylum malabaricum* in an agro-forest landscape. Mol Ecol: 5484–5496. 10.1111/mec.12054 23043256

[13] Ghazoul J (2004) Alien abduction: Disruption of native plant-pollinator interactions by invasive species. Biotropica 36: 156–164.

[14] Munoz AA, Cavieres LA (2008) The presence of a showy invasive plant disrupts pollinator service and reproductive output in native alpine species only at high densities. J Ecol 96: 459–467.

[15] Aizen MA, Ashworth L, Galetto L (2002) Reproductive success in fragmented habitats: do compatibility systems and pollination specialization matter? J Veg Sci 13: 885–892.

[16] Ashworth L, Aguilar R, Galetto L, Aizen MA (2004) Why do pollination generalist and specialist plant species show similar reproductive susceptibility to habitat fragmentation? J Ecol 92: 717–719.

[17] Bond WJ (1994) Do mutualisms matter? Assessing the impact of pollinator and disperser disruption on plant extinction. Philosophical Transactions of the Royal Society of London, Series B 344: 83–90.

[18] Kueffer C, Daehler CC, Torres-Santana CW, Lavergne C, Meyer J-Y, et al (2010) A global comparison of plant invasions on oceanic islands. Perspect Plant Ecol Evol Syst 12: 145–161.

[19] Kaiser-Bunbury CN, Valentin T, Mougal J, Matatiken D, Ghazoul J (2011) The tolerance of island plant-pollinator networks to alien plants. J Ecol 99: 202–213.

[20] Finger A, Kettle CJ, Kaiser-Bunbury CN, Valentin T, Doudee D, et al (2011) Back from the brink: potential for genetic rescue in a critically endangered tree. Mol Ecol 20: 3773–3784. 21883581 10.1111/j.1365-294X.2011.05228.x

[21] Finger A, Kettle CJ, Kaiser-Bunbury CN, Valentin T, Mougal J, et al (2012) Forest fragmentation genetics in a formerly widespread island tree: *Vateriopsis seychellarum* (Dipterocarpaceae). Mol Ecol 21: 2369–2382. 22463385 10.1111/j.1365-294X.2012.05543.x

[22] Kettle CJ, Hollingsworth PM, Jaffre T, Moran B, Ennos RA (2007) Identifying the early genetic consequences of habitat degradation in a highly threatened tropical conifer, *Araucaria nemorosa* Laubenfels. Mol Ecol 16: 3581–3591. 17845432 10.1111/j.1365-294X.2007.03419.x

[23] Ismail S, Huber MJ, Mougal J (2011) *Glionnetia sericea* The IUCN Red List of Threatened Species. Version 2014.2. <www.iucnredlist.org>. Downloaded on 14 September 2014.

[24] Diels L (1922) Beiträge zur Erkenntnis der Vegetation und der Flora der Seychellen. Wissenschaftliche Ergebnisse der Deutschen Tiefsee-Expedition auf dem Dampfer “Valdivia” Zweiter Band, Erster Teil, Dritte Lieferung IV Gustav Fischer Verlag, Jena, Germany: 1898–1899.

[25] Daehler CC, Denslow JS, Ansari S, Kuo HC (2004) A risk-assessment system for screening out invasive pest plants from Hawaii and other Pacific Islands. Conserv Biol 18: 360–368.

[26] Fleischmann K (1997) Invasion of alien woody plants on the islands of Mahe and Silhouette, Seychelles. J Veg Sci 8: 5–12.

[27] Finger A, Kaiser-Bunbury CN, Kettle CJ (2011) Development of polymorphic microsatellite markers of the Seychelles endemic tree *Glionnetia sericea* (Rubiaceae). Conserv Genet Res DOI:10.1007/s12686-011-9515-3.

[28] Peakall R, Smouse PE (2006) GENALEX 6: genetic analysis in Excel. Population genetic software for teaching and research. Mol Ecol Notes 6: 288–295. 10.1093/bioinformatics/bts460 PMC3463245 22820204

[29] Goudet J (1995) FSTAT (Version 1.2): A computer program to calculate F- statistics. J Hered 86: 485–486.

[30] Excoffier L, Laval G, Schneider S (2005) Arlequin (version 3.0): An integrated software package for population genetics data analysis. Evol Bioinform 1: 47–50. PMC2658868 19325852

[31] Pritchard JK, Stephens M, Donnelly P (2000) Inference of population structure using multilocus genotype data. Genetics 155: 945–959. 10835412 10.1093/genetics/155.2.945 PMC1461096

[32] Evanno G, Regnaut S, Goudet J (2005) Detecting the number of clusters of individuals using the software STRUCTURE: a simulation study. Mol Ecol 14: 2611–2620. 15969739 10.1111/j.1365-294X.2005.02553.x

[33] Kalinowski ST, Taper ML, Marshall TC (2007) Revising how the computer program CERVUS accommodates genotyping error increases success in paternity assignment. Mol Ecol 16: 1099–1106. 17305863 10.1111/j.1365-294X.2007.03089.x

[34] Marshall TC, Slate J, Kruuk LEB, Pemberton JM (1998) Statistical confidence for likelihood-based paternity inference in natural populations. Mol Ecol 7: 639–655. 9633105 10.1046/j.1365-294x.1998.00374.x

[35] Ritland K (2002) Extensions of models for the estimation of mating systems using n independent loci. Heredity 88: 221–228. 11920127 10.1038/sj.hdy.6800029

[36] Dafni A, Maués MM (1998) A rapid and simple procedure to determine stigma receptivity. Sexual Plant Rreproduction 11: 177–180.

[37] R Core Team (2014) R: A language and environment for statistical computing. R Foundation for statistical computing, Vienna, Austria. ISBN 3-900051-07-0. Available: http://www.R-project.org/.

[38] Bolker BM, Brooks ME, Clark CJ, Geange SW, Poulsen JR, et al (2009) Generalized linear mixed models: a practical guide for ecology and evolution. Trends Ecol Evol 24: 127–135. 19185386 10.1016/j.tree.2008.10.008

[39] Bates D (2005) Fitting linear mixed models in R. R News: 27–30.

[40] Chybicki IJ, Burczyk J (2009) Simultaneous Estimation of Null Alleles and Inbreeding Coefficients. J Hered 100: 106–113. 18936113 10.1093/jhered/esn088

[41] Angeloni F, Ouborg NJ, Leimu R (2011) Meta-analysis on the association of population size and life history with inbreeding depression in plants. Biol Conserv 144: 35–43.

[42] Fleischer-Dogley F, Kettle CJ, Edwards PJ, Ghazoul J, Määttänen K, et al (2011) Morphological and genetic differentiation in populations of the dispersal-limited coco de mer (Lodoicea maldivica): implications for management and conservation. Divers Distrib 17: 235–243.

[43] Linhart Y, Mendenhall J (1977) Pollen dispersal by hawkmoths in a *Lindenia rivalis* Benth. population in Belize. Biotropica 9: 143.

[44] Bawa KS (1990) Plant-pollinator interactions in tropical rain forests. Annu Rev Ecol Syst 21: 399–422.

[45] Janzen DH (1994) Priorities in tropical biology. Trends Ecol Evol 9: 365–367. 21236892 10.1016/0169-5347(94)90053-1

[46] Haber WA, Frankie GW (1989) A tropical hawkmoth community - Costa Rican dry forest Sphingidae. Biotropica 21: 155–172.

[47] Chase MR, Moller C, Kesseli R, Bawa KS (1996) Distant gene flow in tropical trees. Nature 383: 398–399.

[48] Lourmas M, Kjellberg F, Dessard H, Joly HI, Chevallier MH (2007) Reduced density due to logging and its consequences on mating system and pollen flow in the African mahogany *Entandrophragma cylindricum* . Heredity 99: 151–160. 17473865 10.1038/sj.hdy.6800976

[49] Bittencourt JVM, Sebbenn AM (2007) Patterns of pollen and seed dispersal in a small, fragmented population of the wind-pollinated tree *Araucaria angustifolia* in southern Brazil. Heredity 99: 580–591. 17928810 10.1038/sj.hdy.6801019

[50] Liepelt S, Bialozyt R, Ziegenhagen B (2002) Wind-dispersed pollen mediates postglacial gene flow among refugia. Proc Natl Acad Sci USA 99: 14590–14594. 12391327 10.1073/pnas.212285399 PMC137927

[51] Moulds MS (1981) Larval Food Plants Of Hawk Moths Lepidoptera Sphingidae Affecting Commercial Crops In Australia. General and Applied Entomology 13: 69–80.

[52] Morales C, Traveset A (2009) A meta-analysis of impacts of alien vs. native plants on pollinator visitation and reproduction success of co-flowering native plants. Ecol Lett 12: 716–728. 19453616 10.1111/j.1461-0248.2009.01319.x

[53] Traveset A, Richardson D (2006) Biological invasions as disruptors of plant reproductive mutualisms. Trends Ecol Evol 21: 208–216. 16701087 10.1016/j.tree.2006.01.006

